# Direct-Acting Oral Anticoagulants in patients at extremes of body weight: a review of pharmacological considerations and clinical implications

**DOI:** 10.1055/s-0043-1776989

**Published:** 2024-01-08

**Authors:** Rosa Talerico, Roberto Pola, Frederikus Albertus Klok, Menno Volkert Huisman

**Affiliations:** 1 Section of Internal Medicine and Thromboembolic Diseases, Department of Internal Medicine, Fondazione Policlinico Universitario A. Gemelli IRCCS, Università Cattolica del Sacro Cuore, Rome, Italy; 2 IRCCS San Raffaele, Rome, Italy; 3 Department of Medicine—Thrombosis and Hemostasis, Leiden University Medical Center, Leiden, The Netherlands.

**Keywords:** direct-acting oral anticoagulants, obese, underweight, extremes of body weight, pharmacokinetics

## Abstract

Patients at extremes of body weight are underrepresented in randomized controlled trials of direct-acting oral anticoagulants (DOACs). Therefore, their optimal anticoagulant treatment remains a topic of debate.

The aim of this narrative review is to summarize the evidence on the pharmacokinetic and pharmacodynamic profile of DOACs for treating patients at extremes of body weight in venous thromboembolism (VTE) and in the prevention of cardioembolic stroke in nonvalvular atrial fibrillation (NVAF). A literature search was conducted in the main bibliographic databases, and the most relevant reviews and original articles on the topic were selected.

Although data in these patient groups are limited, apixaban and rivaroxaban show a favorable pharmacokinetic and pharmacodynamic profile in obese VTE treatment and NVAF patients and, in the case of apixaban, also in underweight patients. In particular, these drugs demonstrated comparable efficacy and safety to standard therapy. Very few data were available for dabigatran and edoxaban; the latter drug was safer at a lower dose, mainly in underweight patients.

Our findings are in line with the last International Society of Haemostasis and Thrombosis position paper and European Heart Rhythm Association 2021 practical guide, suggesting the use of apixaban and rivaroxaban in morbidly obese patients (>120 kg or body mass index ≥40 kg/m ^2^ ) and the reduced dosage of edoxaban in low-weight patients. Future studies should focus on large populations of patients at extremes of body weights to acquire more clinical and pharmacokinetic evidence on all available DOACs, especially those currently less investigated.

## Introduction

Direct-acting oral anticoagulants (DOACs) have been approved for the treatment and prophylaxis of venous thromboembolism (VTE) 1 2 3 and for the prevention of cardioembolic stroke in patients with nonvalvular atrial fibrillation (NVAF), 4 5 and have become the first-choice antithrombotic treatment over vitamin K antagonists (VKAs). Based on results from phase III randomized controlled trials (RCTs), DOACs offer a favorable safety profile and more reliable therapeutic effect without the need for regular monitoring and fewer drug interactions than VKA. 6 These agents are administered once-daily (OD) or twice-daily (BID), with fixed dosage determined by indication, age, creatinine clearance, body weight (BW), and the use of concomitant drugs. 7 In addition, DOACs have a predictable pharmacokinetic and pharmacodynamic profile; therefore, routine coagulation monitoring is not required. 8 At the same time, the safety and efficacy of DOACs in patients at extremes of BW is subject of debate. Indeed, the effects of BW on the pharmacokinetic and pharmacodynamic profile of these drugs have led to the hypothesis that in morbidly obese patients a fixed dose of DOACs might not be sufficient due to higher volumes of distribution, whereas in underweight patients, a fixed dose might increase the risk of bleeding due to small volumes of distribution. 9

**Fig. 1:**
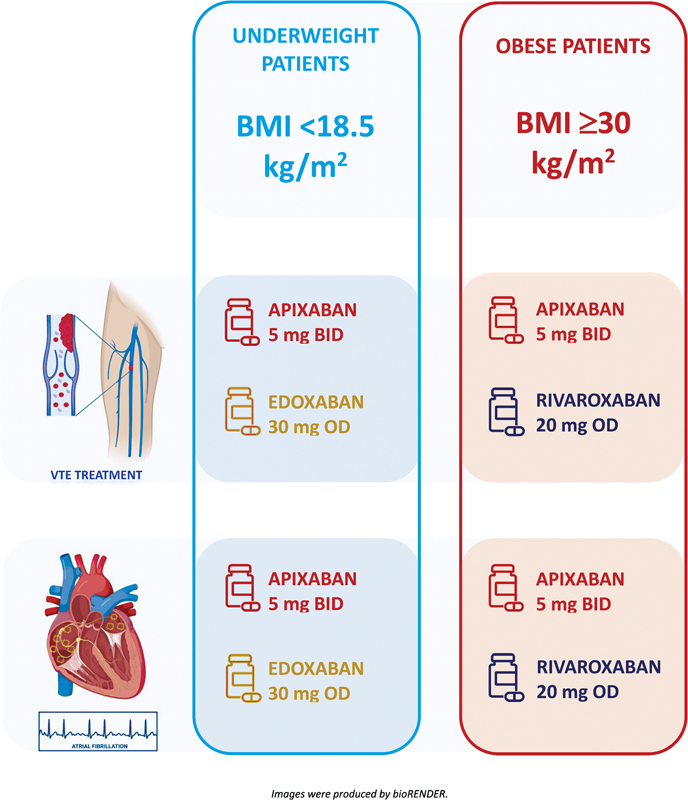
**DOACs in patients at extremes of body weight.** BID, twice-daily; DOACs, direct-acting oral anticoagulants; OD, once-daily; VTE, venous thromboembolism.

Published data on the topic are sparse as only a limited number of obese and underweight patients (extremes of BWs) were enrolled in the phase III RCTs. The results of these trials can therefore not be directly translated to patients at extremes of weight. The aim of this narrative review was to assess efficacy and safety of DOACs and to summarize recent evidence on the pharmacokinetic and pharmacodynamic alterations of DOACs used for the treatment and prevention of VTE recurrence and for the prevention of cardioembolic stroke in patients at extremes of BW ( Fig. 1 ).

## Direct-Acting Oral Anticoagulants in Obese and underweight Patients: Why Does It Concern Us?

Overweight and obesity are two conditions characterized by abnormal and excessive fat or adipose tissue in the body. Globally, more than 1.9 billion adults were reported to be overweight and over 650 million were obese in 2016. 10 According to the World Health Organization, overweight is defined as a body mass index (BMI) ≥25 kg/m ^2^ , obesity as a BMI ≥30 kg/m ^2^ , and morbid or severe obesity as a BMI of ≥40 kg/m ^2^ .

Obesity is a well-established cardiovascular risk factor, associated with increased rates of VTE 11 and atrial fibrillation. 12 The Multiple Environmental and Genetic Assessment study, a Dutch population-based case cohort study involving 3,834 cases and 4,683 controls, revealed that people with BMI ≥30 kg/m ^2^ had a twofold to threefold increased risk of VTE compared with controls with a normal BMI. 13 In addition, obesity (BMI ≥30 kg/m ^2^ ) is a factor predicting VTE recurrence in the validated prediction model “ *MEN continues and HERDOO2.* ” 14 Obesity increases the risk of developing NVAF by 49% in the general population and the risk escalates in parallel with increasing BMI. 12

Although underweight patients are numerically fewer than obese patients, as shown by data from the Centers for Disease Control and Prevention, 15 their prevalence in 2015 to 2016 was calculated to be 1.5% of the adult population. Underweight, defined as a BMI less than 18.5 kg/m, 2 15 is a condition frequently associated with other comorbidities (i.e., older age, kidney failure), increasing the susceptibility to potential adverse events. 16

To support physicians in their choices, the 2021 European Heart Rhythm Association (EHRA) practical guide 17 and a position paper published by the International Society of Haemostasis and Thrombosis (ISTH) 18 have provided advice on the use of DOACs also in special populations. The 2021 EHRA guide 17 states that all DOACs appear to be safe and effective up to a BMI of 40 kg/m ^2^ , whereas data are less robust for a BMI ≥40 kg/m ^2^ ; for patients with BMI ≥50 kg/m ^2^ , measurement of plasma levels during DOAC or conversion to VKA therapy is suggested to be reasonable. The guidance statement from ISTH 18 for use of DOACs in patients with obesity concludes that the use of any DOAC is appropriate for patients with BMI up to 40 kg/m ^2^ or a BW of 120 kg. In patients with a BMI >40 kg/m ^2^ or weighing >120 kg, it is suggested to use the standard doses of apixaban or rivaroxaban for VTE treatment and prevention regardless of BMI and BW, while data for dabigatran, edoxaban, and betrixaban are largely unavailable.

## Search Strategy

A search was performed on PubMed, Cochrane, Embase, and Web of Science databases covering the period until April 7, 2022. Main concepts searched were “DOAC,” “obesity,” “underweight,” “pharmacokinetics,” and “pharmacodynamics.” Duplicate records were removed, and studies were identified using a combination of each of the databases' unique subject headings and keywords, when applicable.

For study selection we considered prospective observational studies as well as systematic and narrative reviews 19 20 21 22 23 (based on RCTs, prospective or retrospective observational cohort studies, and case–control studies) in obese (BW >120 kg and/or BMI >40 kg/m ^2^ ) and underweight (BW <60 kg and/or BMI <18 kg/m ^2^ ) adult patients who received DOACs or VKAs for the treatment of NVAF or VTE. We excluded studies published only as abstracts and non-peer-reviewed and preliminary documents. In addition, further references were identified from a review of references of identified relevant papers. Based on these criteria, 549 articles were identified. From the titles, 108 articles were selected. After reading the abstracts, 69 articles were considered. Finally, 43 articles were included.

Efficacy of the DOAC therapy was interpreted as the occurrence of ischemic stroke or systemic arterial embolism in the NVAF population and recurrent VTE in the VTE population, respectively. Safety was evaluated by assessing the incidence of bleeding. The most commonly used definition to describe major bleedings (MBs) and clinically relevant nonmajor bleedings (CRNMBs) was that of ISTH. 24

## DOACs for VTE Treatment and Secondary Prevention in Obese Patients

### Rivaroxaban

In a post-hoc analysis of the EINSTEIN deep vein thrombosis (DVT) and pulmonary embolism (PE) trials, 25 which included 861 patients with BMI ≥35 kg/m ^2^ , no differences in VTE recurrence were found between rivaroxaban and warfarin [3.0 vs. 2.1% (hazard ratio [HR]: 1.45; 95% confidence interval [CI]: 0.62–3.39)], including those ≥120 to 140 kg and ≥140 kg, with overall low event rates. MB events were numerically lower in patients treated with rivaroxaban versus standard therapy across all categories of BW and BMI, also in patients with BMI ≥35 kg/m ^2^ (5.0 vs. 7.0% [HR: 0.71; 95% CI: 0.22–2.24]). The authors concluded that a high BMI was not associated with an increased risk of recurrent VTE during anticoagulation, although the number of patients with BW ≥140 kg ( *n* = 81) was relatively small.

A prospective observational study 26 was conducted in 167 patients divided in three weight groups (<50 kg: *n* = 18, 50–120 kg: *n* = 105 and >120 kg: *n* = 44). Rivaroxaban plasma levels were measured and bleeding episodes and recurrent VTE rates during follow-up period were assessed. The authors found that patients with weight >120 kg or BMI ≥40 kg/m ^2^ had rivaroxaban levels comparable to those with a standard BW. Notably, they observed a slightly higher rate of recurrent thrombosis (3.0%) compared with larger phase III clinical studies (2.1% in Einstein DVT 27 and PE studies 28 ), with a similar rate of MB (1.1%) compared with a post-hoc analysis of the Einstein DVT and PE studies 29 (1.0%). The reason for the higher rate of recurrent thrombosis observed in this study may be related to a relatively small patient cohort.

Another prospective observational study 30 involved obese patients (BMI ≥30 kg/m ^2^ ) with VTE, in whom rivaroxaban and apixaban concentrations were measured at different time points after intake. These were compared with published reference values for nonobese patients and correlated to incident bleeding and thrombosis events during follow-up. Out of the 146 patients, 22 presented with DOAC concentrations outside the on-therapy ranges: 19/22 with a BMI varying from 31 to 42 kg/m ^2^ had lower than expected DOAC concentrations. Most (14/19) of these patients were treated with rivaroxaban, and the others (5/22) with apixaban. Only 3/22 patients with a BMI between 35.9 and 42.9 kg/m ^2^ had concentrations higher than the usual on-therapy ranges (all rivaroxaban). During follow-up, two patients had a recurrent VTE. No patient had a MB, but 11 patients suffered a minor bleeding. Among these patients, 1/11 with a BMI of 46.9 kg/m ^2^ had a rivaroxaban concentration slightly above the on-therapy ranges, while all others had DOAC concentrations within the expected value.

A third observational study 31 was conducted in the United Kingdom using FIRST registry data and focused on patients with a BMI >40 kg/m ^2^ or BW >120 kg, who received rivaroxaban in the standard dose for acute VTE: 97 participants were recruited, of which 11 had a BW >150 kg. The primary clinical outcomes were objectively diagnosed recurrent VTE, MB or CRNMB, and all-cause mortality. During follow-up, no recurrent VTE or MB diagnoses were reported, and six patients were diagnosed with CRNMB.

The EINSTEIN Investigators 27 performed a randomized study that compared rivaroxaban alone (20 mg OD) with placebo for an additional 6 or 12 months in patients who had completed treatment for acute VTE. A subgroup analysis was conducted, and patients were divided by BW (≤70, 70–90, >90 kg) but not by BMI. Patients weighing >90 kg accounted for approximately 31% in the rivaroxaban group and 30% in the placebo group. There were 0.5% versus 10.2% thrombotic events (composite of DVT or nonfatal or fatal PE) and 7.6% versus 0.6% bleeding events (MB and CRNMB) in the rivaroxaban versus placebo groups, respectively.

Concerning the use of rivaroxaban in the extended treatment of VTE at the dosage of 10 mg OD or 20 mg OD versus aspirin 100 mg OD (EINSTEIN CHOICE trial 32 ), data on obese patients are limited. A subgroup analysis conducted in patients with a BMI ≥30 kg/m ^2^ showed the following primary composite outcome rates (fatal and nonfatal VTE): 1.6% on rivaroxaban 10 mg OD, 1.3% on rivaroxaban 20 mg OD, and 3.7% on aspirin 100 mg OD, whereas they were 0.9, 1.7, and 4.8% in patients with BMI <30 kg/m ^2^ , respectively. Regarding the composite safety outcome (MB and CRNMB), a subgroup analysis was not conducted according to BMI greater or less than 30 kg/m ^2^ . In addition, a subgroup analysis of the primary efficacy outcome by BW (≤70, 70–90, ≥90 kg) in patients ≥90 kg, the following events occurred: 7/396 (1.8%) on rivaroxaban 20 mg OD, 5/390 (1.3%) on rivaroxaban 10 mg OD, and 14/395 (4.9%) on aspirin 100 mg OD. With regard to the primary safety outcome, the events were as follows: 11/360 (3.1%) on rivaroxaban 20 mg OD, 9/365 (2.5%) on rivaroxaban 10 mg OD, and 6/346 (1.7%) on aspirin 100 mg OD.

Regarding the pharmacokinetic aspect, a trial conducted in healthy volunteers divided by BW groups (low [≤50 kg], reference range [70–80 kg], and high [>120 kg]) on rivaroxaban 10 mg OD therapy 33 showed that the observed plasma concentration ( *C*_max_ ) was unaffected in subjects >120 kg but was increased by 24% in subjects weighing ≤50 kg. The bioavailability of rivaroxaban, in terms of area under the concentration–time curve extrapolated to infinity (AUC _(0, ∞)_ ) of plasma concentrations, was similar in all three weight categories. The authors concluded that rivaroxaban is unlikely to require dose adjustment based on BW.

### Apixaban

Data from RCTs, specifically from AMPLIFY trial, 34 showed that 19.4% of patients in the apixaban group weighed 100 kg or more, and 13% had a BMI of >30 kg/m ^2^ .

A post-hoc analysis of this trial 35 evaluated the efficacy, safety, and exposure of apixaban compared with standard therapy by BW and BMI categories (5,384 and 5,359 patients, respectively). In patients with a BW >120 kg ( *n* = 290), the relative risk (RR) (95% CI) of recurrent VTE or VTE-related death was 0.20 (0.02–1.72), the RR (95% CI) of MB was 0.34 (0.04–3.22), and the RR (95% CI) of MB or CRNMB was 0.28 (0.12–0.66). In patients with BMI >40 kg/m ^2^ ( *n* = 263), the RR (95% CI) of recurrent VTE or VTE-related death was 0.55 (0.14–2.15), the RR (95% CI) of MB was 0.27 (0.03–2.40), and the RR (95% CI) of MB or CRNMB was 0.32 (0.12–0.84).

In an observational study published in 2021 of combined data from U.S. insurance claims databases, 36 apixaban compared with warfarin was associated with a lower risk of recurrent VTE (5.3 vs. 8.1 per 100 person-years [HR: 0.63; 95% CI: 0.52–0.78]) and MB (4.5 vs. 6.2 per 100 person-years [HR: 0.70; 95% CI: 0.56–0.89]) in patients with BMI >40 kg/m ^2^ or BW >120 kg.

In the observational study, 30 already mentioned above, patients treated with apixaban were found to have less variation in concentration–time profiles and the peak-to-trough ratio than rivaroxaban.

A recent observational cohort study 37 was conducted in VTE and/or NVAF patients with a BMI >40 kg/m ^2^ and/or a weight >120 kg, treated with apixaban in the usual doses. Anti-Xa levels were routinely measured. The aim of the study was to determine whether BMI or weight was a determinant of anti-Xa levels in this population. Of the 55 patients enrolled, 5 (9%) had peak anti-Xa levels below the reference range and 3 (6%) had trough anti-Xa levels below the reference range. BMI did not correlate with peak or trough anti-Xa levels ( *r* = −0.10, *p* = 0.45 and *r* = −0.14, *p* = 0.31). Weight had a moderate, negative correlation with peak anti-Xa levels ( *r* = −0.42, *p* = 0.002) and a weak, negative correlation with trough anti-Xa levels ( *r* = −0.32, *p* = 0.02). These results are quite in line with a previous analysis conducted on 54 healthy volunteers, 38 divided by BW groups ((low (50 kg), reference (65–85 kg), and high (120 kg)), on apixaban 10 mg OD therapy. Compared with the reference BW group, the high BW group had a *C*_max_ and an AUC _(0, ∞)_ 31% and 23% lower, while the low BW group had a *C*_max_ and an AUC _(0,∞)_ 27% and 20% higher. Plasma anti-factor Xa activity showed a direct, linear relationship with apixaban plasma concentration, regardless of BW group, so the authors concluded that no dose adjustment is recommended for apixaban based on BW alone.

For the extended VTE treatment with apixaban, the AMPLIFY-EXT trial 39 did not dedicate specific sub-analyses to obese patients, focusing on a classification of the population according to BW greater or less than 60 kg.

### Edoxaban

Data on edoxaban for the treatment of VTE in obese patients are very scarce. In HOKUSAI-VTE, 40 approximately 15% of the included patients had a BW >100 kg. The investigators did not assess the outcomes in the patients with obesity separately.

Regarding continuation of anticoagulant therapy >3 months, efficacy and safety data stratified by BW according to follow-up times are not available.

### Dabigatran

The RE-COVER 41 and RE-COVER-II 42 trials enrolled collectively 832 (16.3%) patients with BW >100 kg, 1,071 (21%) patients with BMI of 30.0 to 34.9 kg/m ^2^ , and 579 (11.3%) patients with BMI >35 kg/m ^2^ .

A pooled analysis stratified by BW (<50, 50–100, >100 kg) and BMI (<25, 25–≤30, 30–≤35, and ≥35 kg/m ^2^ ) was performed, without showing an interaction across BW ( *p* -value for interaction = 0.99) and BMI ( *p* -value for interaction = 0.48) categories for primary efficacy outcome (recurrent symptomatic, objectively confirmed VTE and related deaths). Data on the primary safety outcome (MB events), stratified by BW or BMI, are not available.

For the extended treatment of VTE, dabigatran 150 mg BID was compared versus warfarin (RE-MEDY trial) and versus placebo (RE-SONATE trial). 43 In the RE-MEDY study, there were 299 (20.9%) patients with a BW ≥100 kg in the dabigatran group and 300 (21%) patients in the warfarin group. A subgroup analysis showed the rate of VTE recurrence among patients weighing over and under 100 kg on dabigatran versus warfarin was 2.7% versus 1.0% and 1.6% versus 1.3% ( *p* = 0.555 for interaction), respectively. In the RE-SONATE trial, there were 122 (17.9%) patients with a BW ≥100 kg in the dabigatran group and 300 (18.8%) patients in the placebo group. In a subgroup analysis, the VTE recurrence rate among patients weighing over and under 100 kg on dabigatran versus placebo was 0.8 versus 5.6% and 0.36% versus 5.5%, respectively ( *p* = 0.835 for interaction). None of phase three trial programs reported results separately for patients with a BW >100 kg or BMI of ≥40 kg/m ^2^ .

In a retrospective observational study, 44 39 (29.3%) VTE patients on dabigatran therapy weighing ≥120 kg and 314 (29.5%) weighing <120 kg were followed. VTE recurrence was identified in 2.6% in the first group and 0.9% in the second group (odds ratio [OR]: 2.7; 95% CI: 0.27–26.8).

## DOACs for VTE Treatment and Secondary Prevention in Underweight Patients

### Rivaroxaban

Of the more than 8,271 patients included in the EINSTEIN trials, 27 28 166 (2%) had a BW ≤50 kg. In this subgroup, VTE recurrence at 12 months was 6.7% in the rivaroxaban group versus 2.2% in the enoxaparin/VKA group (HR: 2.47, 95% CI: 0.47–12.89); the incidence of MBs was 1.3% for patients treated with rivaroxaban and 4.4% for patients treated with enoxaparin/VKA patients (HR: 0.24; 95% CI: 0.03–2.20).

As mentioned above, the EINSTEIN Investigators 27 showed that in the subgroup analysis for the primary outcome, 3/135 (2.2%) thrombotic events occurred in the rivaroxaban 20 mg OD group and 11/157 (7.0%) in the placebo group in patients with a BW <70 kg. With regard to the primary safety outcome, 5/134 (3.7%) and 2/157 (1.3%) occurred in the rivaroxaban versus placebo group, respectively.

As already mentioned for obese patients, in the EINSTEIN CHOICE trial 32 a subgroup analysis of the primary safety outcome stratified by BW showed the following events in patients ≤70 kg: 11/276 (4%) in the rivaroxaban 20 mg OD group, 7/283 (2.5%) in the rivaroxaban 10 mg OD group, and 5/277 (1.8%) in the aspirin group, respectively. In addition, a subgroup analysis was conducted on frail patients, considered as age >75 or weight ≤50 kg or creatinine clearance <50 mL/min. In terms of primary composite outcome, the following rates were reported for the rivaroxaban 10 mg OD, rivaroxaban 20 mg OD groups, and aspirin 100 mg OD, respectively: 2.4, 0.7, and 3.9% in frail patients, 0.9, 1.7, and 4.5% in nonfrail patients. Regarding the safety outcome, the rates in the rivaroxaban 10 mg OD and 20 mg OD and the aspirin 100 mg OD groups were as follows: 1.2, 4, and 5.1% in frail patients versus 2.6, 3.1, and 1.5% in nonfrail patients, respectively.

### Apixaban

The above-mentioned post-hoc analysis 35 of the AMPLIFY trial evaluated 476 patients with a low BW (≤60 kg) and 1,442 patients with a low BMI (≤25 kg/m ^2^ ). Analysis by BW category showed that the RR (95% CI) of recurrent VTE/VTE-related deaths was 0.63 (0.23–1.72), the RR (95% CI) of MB was 0.15 (0.02–1.15), and the RR (95% CI) of composite of MB and CRNMB was 0.46 (0.24–0.89). In line with this, the analysis by BMI showed a RR (95% CI) for recurrent VTE/VTE-related deaths of 1.0 (0.50–1.97), a RR (95% CI) for MB of 0.41 (0.15–1.18), and a RR (95% CI) of the composite of MB and CRNMB of 0.58 (0.38–0.86). In addition, plasma concentrations of apixaban were measured in a total of 281 patients (16, 199, 45, and 21 patients in the ≤60, >60 to <100, ≥100 to 120, and ≥120 kg BW groups). A modest decrease (<30%) in the median expected exposure (2,990, 2,734, 2,239, and 2,006 ng*h/mL) was observed across increasing weight subgroups.

Extended treatment for VTE was evaluated in the AMPLIFY trial 39 with two different dosages of apixaban (2.5 mg BID and 5 mg BID) versus placebo. In a subgroup analysis, conducted in patients weighing <60 kg, the following rates were observed for the primary composite efficacy outcome (symptomatic recurrent VTE and all-cause death): 6.9% in apixaban 2.5 mg BID, 6.8% in apixaban 5 mg BID, and 18.8% in the placebo group, respectively. In terms of composite safety outcomes (MB and CRNMB), the following rates were observed: 5.2% in apixaban 2.5 BID, 12.1% in apixaban 5 mg BID, and 2.1% in placebo group, respectively.

In terms of composite efficacy outcome, a RR (95% CI) of 1.01 (0.3–3.9) was observed between apixaban 2.5 BID and 5 mg BID, whereas the RR (95% CI) between apixaban 2.5 mg BID and 5 mg BID was 0.5 (0.1–1.7) for the composite safety outcome.

### Dabigatran

Only very few patients with low BW (<50 kg) were included in RE-COVER 41 and RE-COVER II 42 trials (1.1%). As noted above, the pooled analysis of the two trials stratified by BW did not show an interaction across BW categories ( *p* -value for interaction = 0.99) for primary efficacy outcome. In multivariate analysis performed to develop a model to predict MB in the combined data of RE-COVER and RE-COVER II, BW (low or high) was not identified as a relevant predictor. 45 46 Regarding the extended therapy of VTE with dabigatran in underweight patients, in RE-MEDY 43 and RE-SONATE 43 studies only 0.7 and 1.2% respectively of the dabigatran population presented a BW <50 kg. No thrombotic or hemorrhagic events were observed in this population. No pharmacokinetic studies evaluating the use of dabigatran in low-body-weight patients are currently available.

### Edoxaban

The HOKUSAI VTE trial 40 enrolled 524 patients on edoxaban therapy (12.7% of the total edoxaban population) weighing ≤60 kg. A safety or efficacy analysis within the weight categories considered (≤60 kg) was not performed. However, as shown in a subsequent analysis of the trial, 47 733 patients (17.8% of the population) received a reduced edoxaban dose of 30 mg OD, for one of the following reasons: a BW of 60 kg or less ( *n* = 442), creatinine clearance 30 to 50 mL per minute ( *n* = 184), concomitant treatment with potent P-glycoprotein inhibitors ( *n* = 22), or two or more criteria ( *n* = 85). Among patients who qualified for the 30-mg dose of edoxaban, recurrent VTE occurred in 22 of 733 patients (3.0%), as compared with 30 of the 719 patients (4.2%) receiving warfarin (HR: 0.73; 95% CI: 0.42–1.26), while MB or CRNMB occurred in 58/733 (7.9%) of the edoxaban group compared with 92/719 (12.8%) of the warfarin group (HR: 0.62; 95% CI: 0.44–0.86).

MB occurred in 11 patients (1.5%) in the edoxaban group and in 22 patients (3.1%) in the warfarin group (HR: 0.50; 95% CI: 0.24–1.02). Among patients with low BW (≤60 kg), recurrent VTE was reported in 13/442 (2.9%) in the edoxaban group compared with 14/425 (3.3%) in the warfarin group, and MB or CRNMB in 29/442 (6.6%) compared with 51/425 (12.0%). Edoxaban 30 mg OD maintained efficacy and safety with markedly less bleeding than observed in the warfarin group.

Regarding continuation of anticoagulant therapy >3 months, 269/733 patients on edoxaban 30 mg OD (36.7%) and 274/719 (38.1%) on warfarin therapy continued follow-up up to 12 months. Efficacy and safety data stratified by BW according to follow-up times are not available.

## DOACs for Cardioembolic Stroke Prophylaxis in Obese Patients with NVAF

### Rivaroxaban

In a post-hoc analysis of ROCKET AF, 48 the incidence of stroke and systemic embolic events (SEE) as well as bleeding events was compared in normal weight, overweight, and obese patients. The risk of stroke was lower for obese patients with BMI ≥35 kg/m ^2^ than that for normal weight patients in both the rivaroxaban and warfarin groups (rivaroxaban: HR: 0.62, 95% CI: 0.40–0.96; warfarin: HR: 0.48, 95% CI: 0.31–0.74).

Evidence from a large observational study 49 including 35,613 obese patients (BMI ≥30 kg/m ^2^ ) treated with rivaroxaban and 35,613 treated with warfarin reported that rivaroxaban was associated with a reduced risk of stroke and SSE (HR: 0.83, 95% CI: 0.73–0.94) and MB (HR: 0.82, 95% CI: 0.75–0.89) compared with warfarin. Sub-analysis stratified by BMI (30.0–34.9, 35.0–39.9, and ≥40 kg/m ^2^ ) was performed, without showing an interaction across BMI categories for SSE or MB outcomes.

A retrospective cohort study 50 evaluated the effectiveness and safety of rivaroxaban and apixaban in 299 adult patients with BMI ≥50 kg/m ^2^ compared with a cohort of 296 patients with BMI 18 to 30 kg/m ^2^ . The primary endpoint of incidence of ischemic stroke was numerically similar in both groups: 1.3 per 100 patient-years in the BMI ≥50 kg/m ^2^ group compared with 2.0 per 100 patient-years in the BMI <30 kg/m ^2^ group (RR: 0.65, 95% CI: 0.38–1.82). The incidence of MB was 2.1 per 100 patient-years in the BMI ≥50 kg/m ^2^ group versus 3.5 per 100 patient-years in the BMI <30 kg/m ^2^ group (RR: 0.60, 95% CI: 0.34–2.29), while the incidence of CRNMB was 3.6 per 100 patient-years in the obese group, compared with 4.7 per 100 patient-years in the control group (RR: 0.77, 95% CI: 0.55–1.45).

### Apixaban

In a post-hoc analysis of ARISTOTLE trial, 51 the efficacy and safety of apixaban versus warfarin in patients at extremes of BW were evaluated. Of the 18,139 enrolled patients, 982 (5.4%) had a weight >120 kg. In the high-weight category, no difference in the occurrence of efficacy outcomes (stroke or systemic embolism [SE], death from any cause, or myocardial infarction) associated with either apixaban or warfarin was observed. More specifically, the HR for stroke/SE was 0.39 (95% CI: 0.12–1.22). There was a lower risk of MB or CRNMB (HR: 0.58; 95% CI: 0.35–0.95) or any bleeding with apixaban (HR: 0.67; 95% CI: 0.53–0.85) as compared with warfarin. The high-weight group was stratified into two groups: 121 to 140 kg ( *n* = 724 [4%]) and >140 kg ( *n* = 258 [1.4%]). The risk of stroke or SE associated with apixaban was lower than for warfarin in patients weighing 121 to 140 kg (HR: 0.21; 95% CI: 0.05–0.95). In the >140 kg patient cohort, there were only two events in the apixaban group versus one in the warfarin group, making it impossible to compare the two groups. Patients in the 121 to 140 kg weight category treated with apixaban were at comparable risk for MB to those receiving warfarin (HR: 0.99; 95% CI: 0.46–2.10), but had lower rates of any bleeding (HR: 0.61; 95% CI: 0.46–0.80).

### Dabigatran

In the RE-LY trial, 52 3,099/18,113 (17.1%) patients with BW >100 kg were included. An analysis by weight was conducted reporting only an outcome rate per year and the total number of obese patients, without a breakdown of the number of patients in each treatment group. In particular, no differences in rates of stroke or SE were observed between patients randomized to dabigatran 110 mg BID, dabigatran 150 BID, or warfarin (0.8% vs. 0.87% vs. 0.94% [ *p* = 0.49 for the comparison of dabigatran 110 mg with warfarin; *p* = 0.42 for the comparison of dabigatran 150 mg with warfarin]).

In a subsequent pharmacokinetic analysis of RE-LY, 53 plasma concentrations of dabigatran were determined and correlated with the clinical outcomes of ischemic stroke/SE and MB. The population was classified into three groups according to BW (<50 kg, 50–99.9 kg, >100 kg). Patients weighing <50 kg were found to have an 21% higher mean dabigatran concentration than those weighing 50 to 100 kg, and 53% higher than those weighing >100 kg. A multiple logistic regression model (c-statistic 0.66, 95% CI: 0.61–0.71) showed that the risk of ischemic events was inversely related to trough dabigatran concentrations ( *p* = 0.045); however, weight was not found to be a relevant determinant of stroke in logistic regression models.

### Edoxaban

In the ENGAGE AF-TIMI 48 54 trial, 21,028 patients were included, of which 5,209 (24.8%) were moderately obese (BMI 30 to <35 kg/m ^2^ ), 2,099 (10%) severely obese (BMI 35 to <40 kg/m ^2^ ), 1,149 (5.5%) very severely obese (BMI >40 kg/m ^2^ ). Only 148 patients (0.7%) had a BMI >50 kg/m ^2^ . Among severely obese patients, 415 were on edoxaban 60 mg OD, 370 on edoxaban 30 mg OD, and 364 on warfarin. Higher BMI was significantly and independently associated in the whole cohort with lower risks of stroke/SEE (HR: 0.88, 95% CI: 0.82–0.94) and death (HR: 0.91, 95% CI: 0.87–0.95), 55 but with increased risks of MB (HR: 1.06, 95% CI: 1.01–1.12). Specifically, among patients with BMI >40 kg/m ^2^ in the edoxaban 60 mg group compared with warfarin, stroke/SEE occurred in 9 (0.8%) versus 5 (0.5%) (HR: 1.37, 95% CI: 0.37–5.05), and MB in 28 (2.9%) versus 30 (3.5%; HR: 0.92, 95% CI: 0.54–1.57). On the other hand, among patients with BMI >40 kg/m ^2^ in the edoxaban 30 mg group compared with warfarin, stroke/SEE occurred in 12 (1.2%) versus 5 (0.5%; HR: 2.01, 95% CI: 0.58–6.97), and MB in 15 (1.7%) versus 30 (3.5%; HR: 0.47, 95% CI: 0.26–0.85). 55 There were no differences across BMI categories in anti-factor Xa activity and edoxaban trough plasma levels (blood samples were collected 1 month after randomization).

Further evidence on edoxaban comes from the sub-analysis of ETNA-AF-Europe, a multicenter observational study. 56 The objective was to analyze clinical outcomes in 13,092 patients on edoxaban therapy across a range of BW categories (>100 kg, ≤60 kg) as compared with the reference BW group (>60–≤80 kg). There were 1,310, 5,565, 4,346, and 1,446 patients in the ≤60 kg, >60–≤80 kg, >80–≤100 kg, and >100 kg weight groups, respectively. The rates of stroke/SE and MB were low at 1 year, and comparable between the weight groups. Specifically, for patients with BW >100 kg, the annualized event rate for stroke/SE was 0.57% (HR: 0.64, 95% CI: 0.30–1.34). A lower risk of MB (HR: 0.34, 95% CI: 0.15–0.78) and MB or CRNMB (HR: 0.58, 95% CI: 0.37–0.92) was found in patients weighing >100 kg compared with the other weight groups. However, it was no longer observed after adjustment for estimated glomerular filtration rate and CHA _2_ DS _2_ -VASc score (HR: 0.67, 95% CI: 0.27–1.66; HR: 0.98, 95% CI: 0.57–1.69).

## Evidence of Use of DOACs for Cardioembolic Stroke Prophylaxis in Underweight Patients with NVAF

A recent meta-analysis 20 conducted on results from RCTs and a few observational studies and performed including appropriately prescribed DOACs showed that in NVAF patients with low BW ≤60 kg, the use of DOACs was associated with was associated with significantly lower stroke/SE (RR: 0.63, 95% CI: 0.54–0.73; *I* ^2^ : 7%) and MB risks (RR: 0.70, 95% CI: 0.61–0.80; *I* ^2^ : 0%), but similar risk of mortality (RR: 0.71, 95% CI: 0.45–1.11; *I* ^2^ : 90%), compared with warfarin.

### Rivaroxaban

Only 4.62% patients with low BW (identified as ≤70 kg) and 4.25% with BMI ≤25 kg/m ^2^ were included in the ROCKET AF 57 trial. No specific analysis of these subgroups was conducted.

A retrospective study 58 in underweight Korean patients (<60 kg) focused on six clinical outcomes of effectiveness and safety of DOACs ( *n* = 14,013 patients) and warfarin ( *n* = 7,576 patients). DOACs were associated with lower risks of ischemic stroke (HR: 0.59; 95% CI: 0.51–0.69) and MB (HR: 0.71; 95%: CI 0.60–0.83), compared with warfarin. Of them, 6,044 patients were on rivaroxaban therapy. More specifically, 4,295 patients (2,320 on reduced dosage—15 mg OD—and 1,975 on regular dosage—20 mg OD) weighed between 50 and 60 kg, while 1,749 (1,089 on reduced dosage and 660 on regular dosage) weighed <50 kg. HR (95% CI) of ischemic stroke in rivaroxaban therapy compared with warfarin in patients weighing 50 to 60 kg was 0.58 (0.46–0.72), and in the category <50 kg 0.84 (0.70, 0.99). Regarding hospitalization for MB, the HRs (95% CI) in rivaroxaban therapy compared with warfarin were 0.82 (0.60–1.10) for the 50 to 60 kg subgroup, and 1.14 (0.80–1.61) for the <60 kg subgroup.

### Apixaban

A post-hoc analysis of ARISTOTLE 51 assessed the randomized treatment effect stratified by BW (≤60, >60–120, >120 kg). Of 18,139 patients included, 1,985 (10.9%) had a BW ≤60 kg and of these 27% were taking a reduced dose of apixaban (2.5 mg BID). The hazards for stroke/SE in the low-weight group (≤60 kg) were favorable for apixaban-treated patients (HR: 0.6; 95% CI: 0.4–0.9). Apixaban was associated with a lower MB as well (HR: 0.55; 95% CI: 0.36–0.82). No pharmacokinetic and pharmacodynamic data were provided.

### Dabigatran

Only 2% of the total RE-LY 52 population had a low BW (defined as <50 kg) and the study was not powered to identify differences in safety or efficacy in the subgroup of underweight patients.

However, a meta-analysis 9 conducted on a population categorized by BMI classes evaluated the impact of weight on the efficacy and safety of DOACs, showing a trend to a higher incidence of MB with high-dose dabigatran (considered only the dosage of 150 mg BID) in low BMI patients (18.5–25 kg/m ^2^ ) (OR: 1.26; 95% CI: 0.76–2.09). About stroke or systemic SE, instead, the ORs (95% CI) for dabigatran 150 mg and dabigatran 110 mg BID were 0.45 (0.17–1.22) and 0.53 (0.19–1.46), respectively.

As already reported, a prespecified pharmacokinetic analysis 53 of RE-LY showed that patients weighing <50 kg had a higher mean dabigatran concentration than patients in higher weight classes. However, weight appeared not to be a relevant determinant of stroke in multivariate analysis.

An observational study 59 was conducted in a cohort of patients on dabigatran 110 mg BID to monitor the occurrence of the bleeding complications and to verify the relationship between BMI and MB events. Patients were divided into three subgroups (tertiles) by BMI (tertile 1: ≦23.9 kg/m ^2^ , *n* = 273; tertile 2: 23.9< BMI ≤26.5 kg/m ^2^ , *n* = 290; and tertile 3: >26.5 kg/m ^2^ , *n* = 279). MB occurred in 28 patients (3.3%), of which 17 (60.7%) in the first tertile, 7 (25%) in the second tertile, and 4 (14.3%) in the third. The overall stroke or SE rate was 0.95% ( *n* = 8). Analyzing the factors that predicted hospitalization due to bleeding, higher BMI was found to possibly protect against MB (HR: 0.83, 95% CI: 0.74–0.90).

### Edoxaban

A recent analysis 60 of ENGAGE AF-TIMI 48 trial looked at dynamic pharmacokinetics/pharmacodynamics and clinical outcomes in patients randomized to warfarin, higher dose edoxaban (HDER, 60 mg OD), and lower dose edoxaban (LDER, 30 mg OD) regimens at the extremes of BW. The authors analyzed three BW groups: low BW (LBW: <5th percentile, 55 kg), middle BW (MBW: 45th–55th percentile, 79.8–84 kg), and high BW (HBW: >95th percentile, 120 kg). The risks of SSE were similar between HDER and warfarin for each of the three weight groups, while for LDER versus warfarin, the corresponding HRs and 95% CIs were 1.03 (0.59–1.79); 1.04 (0.65–1.65); and 1.79 (0.65–4.91), respectively ( *p*_int_ = 0.061, *p*_int-trend_ = 0.023). MB was reduced by LDER versus warfarin ( *p*_int_ = 0.061, *p*_int-trend_ = 0.023), with a particularly marked effect in LBW patients (HR: 0.27, 95% CI: 0.13–0.54) and a significant reduction also in MBW patients (HR: 0.66, 95% CI: 0.44–0.99). Both HDER and LDER were associated with lower rates of MB or CRNMB versus warfarin ( *p*_int_ = 0.055, *p*_int-trend_ = 0.032, and *p*_int_ = 0.064, *p*_int-trend_ = 0.026, respectively) with larger reductions in LBW and MBW patients.

In underweight patients (≤60 kg), the efficacy and safety of apixaban, in doses of nonunderweight patients, and mainly reduced dose edoxaban (30 mg OD) were consistent compared with warfarin. Dabigatran showed an increased risk of bleeding, while evidence on rivaroxaban is still limited.

## Clinical Considerations

Regarding treatment of VTE in morbid obese patients (>120 kg or BMI ≥40 kg/m ^2^ ), rivaroxaban and apixaban with doses of nonobese patients may represent two appropriate options in terms of efficacy and safety comparable to warfarin. Data on dabigatran are limited, and lack of clinical and pharmacodynamic evidence on edoxaban does not support its use in this category. In underweight patients (≤60 kg), apixaban in doses of nonunderweight patients and reduced dose edoxaban appear to be effective and safer than warfarin therapy. Very few data are currently available for rivaroxaban and dabigatran.

Concerning secondary prevention of VTE in obese patients (>120 kg or BMI ≥40 kg/m ^2^ ), evidence is very scarce, particularly in the case of apixaban and edoxaban. Few data are available for dabigatran, whereas rivaroxaban at both full and reduced doses seems to be an effective alternative, although safety data are still limited. In underweight patients (≤60 kg) full and reduced dose rivaroxaban and reduced dose apixaban and edoxaban appeared to be effective alternatives to standard therapy or placebo, without a significant increase in the risk of bleeding. Data on dabigatran in this category are limited.

In the context of NVAF, among morbid obese patients (>120 kg or BMI ≥40 kg/m ^2^ ) the efficacy and safety data on rivaroxaban and apixaban with doses of nonobese patients compared with warfarin support their routine use. Edoxaban might be considered, but further evidence is needed to establish the safety profile in this category. No data are available for dabigatran. In underweight patients (≤60 kg), apixaban in doses of nonunderweight patients and mainly reduced dose edoxaban showed consistent efficacy and safety compared with warfarin, while dabigatran revealed an increased risk of bleeding. Evidence on rivaroxaban is limited ( Table 1 ).

**Table 1:** Overview of safety and efficacy of the different DOACs in patients at extremes of body weight for the VTE treatment and secondary prevention and stroke prophylaxis in NVAF

Indication	Population	Drug	Final message
VTE treatment	Obese patients	Rivaroxaban	Efficacy and safety may be comparable to LMWH/VKAs
		Apixaban	Efficacy and safety may be comparable to LMWH/VKAs
		Dabigatran	Limited data
		Edoxaban	Lack of evidence
	Underweight patients	Rivaroxaban	Limited data
		Apixaban	Safety and efficacy may be comparable to LMWH/VKAs
		Dabigatran	Limited data
		Edoxaban	Efficacy may be comparable to LMWH/VKAs, increased safety, especially for the reduced dosage
VTE secondary prevention	Obese patients	Rivaroxaban	Efficacy may be greater than aspirin or placebo, limited data on safety
		Apixaban	Lack of evidence
		Dabigatran	Limited data
		Edoxaban	Lack of evidence
	Underweight patients	Rivaroxaban	Efficacy may be greater than aspirin or placebo, without significantly increased risk of bleeding
		Apixaban	Efficacy may be greater than placebo, without significantly increased risk of bleeding for the reduced dosage
		Dabigatran	Limited data
		Edoxaban	Efficacy may be comparable to LMWH/VKAs, increased safety, especially for the reduced dosage
Cardioembolic stroke prophylaxis in NVAF	Obese patients	Rivaroxaban	Efficacy and safety may be comparable to VKAs
		Apixaban	Efficacy and safety may be comparable to VKAs
		Dabigatran	Limited data
		Edoxaban	Efficacy may be comparable to VKAs, but increased risk of bleeding
	Underweight patients	Rivaroxaban	Limited data
		Apixaban	Safety and efficacy may be comparable to VKAs
		Dabigatran	Limited data on efficacy, but increased risk of bleeding
		Edoxaban	Safety and efficacy may be comparable to VKAs, mainly for reduced dosage

Abbreviations: LMWH/VKA, low-molecular-weight heparin (LMWH) followed by vitamin K antagonists (VKAs); NVAF, nonvalvular atrial fibrillation; VTE, venous thromboembolism.

Note: obese patients: BMI ≥30 kg/m ^2^ ; underweight patients: <18.5 kg/m ^2^ .

It remains a matter of debate whether measuring DOAC plasma levels may help to better manage anticoagulation therapy in patients at extremes of BW. This is a controversial topic, both because of the lack of clear correlation between drug levels, effective anticoagulation, and outcome, as well as because of the variability and uncertainty in the interpretation of results. This uncertainty is demonstrated by contradicting suggestions by current guidance documents. The EHRA 2021 17 Practical Guide suggests to consider DOAC plasma level measurement in patients with a BMI ≥40 kg/m ^2^ , while the ISTH position paper 18 suggests against regular assessment of peak or trough drug-specific DOAC levels. The fact that the studies described in this narrative review used fixed rather than plasma-level adjusted doses of DOACs supports the ISTH point of view.

## Conclusions

Although the evidence on DOACs in patients at extremes of BW is far from conclusive, our findings are in line with the ISTH position paper and the EHRA 2021 practical guide. Current findings suggest that, with regard to the treatment of VTE, apixaban and rivaroxaban, both in doses used in the phase 3 trials, may be prescribed to morbidly obese patients, as well as apixaban and reduced dose edoxaban to underweight patients. With regard to secondary prevention of VTE, data on morbidly obese patients are not yet conclusive, while in underweight patients reduced dosage apixaban and edoxaban and full and reduced dose rivaroxaban may be used. With regard to cardioembolic stroke prophylaxis in patients with NVAF, apixaban and rivaroxaban can be used in morbidly obese patients, both in doses used in the phase 3 trials, as can apixaban and reduced dose edoxaban in the underweight patients. More evidence is needed to establish the safety profile of edoxaban in morbidly obese patients with NVAF. Current data do not allow the use of dabigatran in these extreme categories, neither for the treatment and secondary prevention of VTE nor for cardioembolic stroke prophylaxis in NVAF. To date, few and inconclusive pharmacokinetic data are available for patients at the extremes of BW. Therefore, it is not possible to make conclusive remarks on the pharmacokinetic profile of DOACs in the categories considered.

## Highlights

- Anticoagulant therapy represents a challenge in patients at extremes of body weight.
- The evidence on the use of direct oral anticoagulants (DOACs) in this category is not yet conclusive.
- Some DOACs more than others may be considered in cases of atrial fibrillation or venous thromboembolism (VTE).
- Evidence on the use of DOACs at extremes of body weight in VTE recurrence prevention is summarized for the first time.
